# 

**Published:** 1885-12

**Authors:** 


### BOOKS AND PAMPHLETS RECEIVED.

Report of Calcutta Homœopathic Charitable Dispensary for 1884–85.

The Surgical Treatment of Cysts of the Pancreas. By N. Senn, M. D., Milwaukee, Wis.

Iritis, Its Relation to the Rheumatic Diathesis and its Treatment. By Charles J. Lundy, A. M., M. D., reprinted from September No. of *The Physician and Surgeon.*

Drugs and Medicines of North America. Edited by J. U. and C. J. Lloyd. September No.

